# Atractylenolide I inhibits EMT and enhances the antitumor effect of cabozantinib in prostate cancer *via* targeting Hsp27

**DOI:** 10.3389/fonc.2022.1084884

**Published:** 2023-01-06

**Authors:** Pengfei Qiao, Zhentao Tian

**Affiliations:** ^1^ The Department of Urology Surgery, First Teaching Hospital of Tianjin University of Traditional Chinese Medicine, Tianjin, China; ^2^ National Clinical Research Center for Chinese Medicine Acupuncture and Moxibustion, Tianjin, China

**Keywords:** prostate cancer, ATL-1, proliferation, apoptosis, Hsp27

## Abstract

**Objective:**

To investigate the effect of Hsp27 and the inhibitory effect of Atractylenolide I (ATL-1) on the proliferation of prostate cancer cell DU145 and PC-3.

**Methods:**

MTT assay was used to detect the inhibitory effect of silencing Hsp27 and ATL-1 on DU145 and PC-3 proliferation of prostate cancer cells. TUNEL detected the apoptosis rate of prostate cancer cell DU145 and PC-3 after silencing Hsp27 and ATL-1 treated. qRT-PCR was used to detect the changes of apoptosis related genes caspase-3, PARP, Bax and Bcl-2 in prostate cancer cell DU145 and PC-3 after the effect of silencing Hsp27 and ATL-1 treated. At the same time, the antitumor effect of ATL-1 combined with cabozantinib was analyzed.

**Results:**

Hsp27 was highly expressed in human prostate cancer. MTT assay showed that ATL-1 inhibited the proliferation of prostate cancer cells DU145 and PC-3 compared with the control group. TUNEL results showed that silencing Hsp27 and ATL-1 treated could significantly promote the apoptosis of prostate cancer cells DU145 and PC-3 compared with the control group. qRT-PCR results showed that compared with the control group, ATL-1 could promote the expression of caspase-3, PARP and Bax in DU145 and PC-3 prostate cancer cells. Inhibition of Hsp27 by ATL-1 reduced cell viability and induced apoptosis. ATL-1 inhibits the antitumor effect of Hsp27 - enhanced cabozantinib. Hsp27 regulates eIF4E and mediates cell protection.

**Conclusion:**

Silencing Hsp27 inhibits EMT. ATL-1 can inhibit the malignant evolution of prostate cancer cells by inhibiting Hsp27/eIF4E. ATL-1 also enhanced chemosensitization of cabozantinib in prostate cancer.

## 1 Introduction

Prostate cancer (PCa) is a malignant tumor of the male genitourinary system, which is prone to bone metastasis (1). Potential specific targets are difficult to develop targeted drugs and clinical treatment (2, 3).

Heat shock proteins (HSPs) play an important biological role in prostate cancer as molecular chaperones involved in the pathogenesis, diagnosis, treatment and prognosis of various tumors (4, 5). *Hsp27* is considered to be a biomarker similar to prostate specific membrane antigen (6). Among them, the increased expression of heat shock protein family member Hsp27 is associated with castration resistance of prostate cancer (7). Hsp27 promotes drug resistance, invasion, and bone metastasis, making prostate cancer more difficult to treat (8). Hsp27 is involved not only in the cellular response to heat shock, but also in oxidative or chemical stress (9). Hsp27 is involved in the process of tumor invasion and metastasis, and its function has been paid more and more attention. In hepatocellular carcinoma (HCC), shRNA-mediated Hsp27 silencing can reduce the proliferation, migration and invasion of HCC cells (10). Konda et al. (11) confirmed that oncogene cells require Hsp27 to survive and that Hsp27 may interfere with the effect of targeted therapy. Therefore, targeting Hsp27 in prostate cancer could be a promising tumor therapy strategy.

ATL-1 is a sesquiterpene lactone compound extracted from the rhizome of Atractylodes macrocephala, compositae, with anti-inflammatory and anti-tumor activities (12–16). ATL-1 has a significant inhibitory effect on leukemia, bladder cancer and melanoma (17). Recent studies have shown that ATL-1 can enhance the sensitivity of ovarian cancer to paclitaxel by blocking TLR4/MYD88-dependent signaling pathway (18). However, few studies have been done on the antitumor effect of ATL-1 on prostate cancer, and its molecular mechanism remains unclear. Therefore, this study investigated the molecular mechanism of ATL-1 inhibiting the proliferation of prostate cancer cells.

In this study, the effects of ATL-1 on human prostate cancer cells were studied. To elucidate the mechanism of ATL-1 inhibiting the proliferation of prostate cancer cells by detecting the changes of cell viability and apoptosis. This study provides a basis for the clinical application of ATL-1 and a new idea for the development of other drugs for prostate cancer treatment.

## 2 Methods

### 2.1 Bioinformatics analysis

By using the human protein atlas (http://www.proteinatlas.org/) for the purpose of database selection Hsp27 gene search. The tumor type was prostate cancer. To analyze the expression of Hsp27 in prostate cancer tissues. Meanwhile, single cell sequencing results of Hsp27 in Prostate tissues were analyzed by the human protein atlas (http://www.proteinatlas.org/). Co-expressed genes of Hsp27 were analysed by LinkedOmics (http://www.linkedomics.org/login.php). GO and KEGG functional enrichment analysis of Hsp27 co-expressed genes was further analysed.

### 2.2 Cell culture

Prostate cancer cells DU145 and PC-3 were purchased from American Type Culture Collection (ATCC, Manassas, VA, USA) and inoculated in RPMI-1640 medium containing 10% fetal bovine serum (Gibco, Life Technologies, Rockville, MD, USA). Conventional culture and passage in 37°C, 5% carbon dioxide incubator (Thermo Fisher Scientific, Waltham, MA, USA).

### 2.3 Cell transfection

The cells in the logarithmic growth phase were taken, and the cells were transfected according to the instructions of the Lipofectamine 2000 transfection kit. Cells were divided into: blank group (empty plasmid), Hsp27 overexpression group. DU145 and PC-3 cells were seeded into 6-well plates at 2×10^5^/well. After adding the Hsp27 overexpression plasmid, the medium was changed 6h. After 48 hours of transfection, the cells were washed 3 times with PBS, and the cells were collected by trypsinization. The effect of plasmid transfection was detected by qPCR method.

### 2.4 qRT-PCR

Collect the cells verified by transfection, and extract total RNA in strict accordance with the instructions of RNA extraction and reverse transcription related kits (TaKaRa Biotechnology Co., Ltd., Dalian, China). Quickly reverse-transcribe it into complementary DNA (cDNA). Strictly according to the qRT-PCR instructions, GAPDH was used as the internal reference for detection. Three replicate wells were set for each sample, and the final volume of the reaction system was 20 μL. Among them, 2 μL of reverse transcription product, 10 μL of SYBR Green Mix, 1 μL of forward and reverse primers, and 6 μL of water. The reaction conditions were 95°C for 30 s, 60°C for 30 s, 72°C for 45 s, a total of 40 cycles, and 72°C for 5 min. HSP27, F: 5’-CAGTCCAACGAGATCACCATC-3’ and R; 5’-CAGTCTCATCGGATTTTGCAG-3’. Bcl-2, F: 5’-ATGCCTTTGTGGAACTATATGGC-3’, R: 5’-GGTA TGCACCCAGAGTGATGC-3’. Bax, F: 5’-TGAAGACAGG GGCCTTTTTG-3’, R: 5’-AATTCGCCGGAGACACTCG. E-cadherin, F: 5′-TGCCCAGAAAATGAAAAAGG-3′, R: 5′-GTGTATGTGGCAATGCGTTC-3′. Vimentin, F: 5′-GAG AACTTTGCCGTTGAAGC-3′, R: 5′-GCTTCCTGTA GGTGGCAATC-3′. GAPDH, F: 5’-GGTGAAGGTCGGAGT CAACG-3’, R: 5’-CAAAGTTGTCATGGATGCACC-3’. The relative expression of target gene mRNA was detected by 2^-ΔΔCt^ method. The experiment was performed 3 times.

### 2.5 Determined by MTT test

After 72 h culture, add 20 μL MTT (5 g/L) (Beyotime, Shanghai, China) solution to each well, respectively. The supernatant was removed after 4 h incubation in the incubator, and 150 μL DMSO (Sigma-Aldrich, St. Louis, MO, USA) was added to each well. The precipitation was dissolved by shaking for 10 min at room temperature, and the absorbance at 490 nm was detected by a microplate reader. Cell proliferation activity (%) = experimental group absorbance/blank control group absorbance ×100% (MultiskanEX, Lab systems, Helsinki, Finland).

### 2.6 TUNEL experiment

The cells were inoculated on polylysine-coated cell glass coverslips at a density of 1×10^6^ cells/mL. Incubate for 24 h under constant temperature and humidity. After treatment, it was fixed with 4% paraformaldehyde at room temperature for 30min. Incubate with H_2_O_2_ in methanol for 15 min. Next, 0.1% Triton X-100 sodium citrate permeable solution was incubated for 10 min at 2-8°C. After incubation, TUNEL staining solution (Beyotime, Shanghai, China) was prepared according to the proportion of TdT (1μL) and fluorescein labeled dUTP solution (9 μL). Then, 50 μL TUNEL staining solution was added to each sample. After incubation at 37°C for 1h, each sample was added with 30 μL DAPI staining solution for 5 min. After the cells were washed with PBS, they were observed under fluorescence microscope under dark conditions. The apoptotic cells showed blue-green fluorescence after staining. The proportion of apoptotic cells in each group was calculated and analyzed by the statistical method.

### 2.7 Statistical analysis

SPSS 20.00 software (SPSS Inc., Chicago, IL, USA) was used for statistics. Measurement data were expressed as mean ± standard deviation. Comparison between the two groups was performed by two independent sample T test. Comparison between multiple groups was performed by one-way ANOVA. *p*<0.05 was considered as statistically significant difference.

## 3 Results

### 3.1 Expression of Hsp27 in prostate cancer and functional enrichment analysis of its co-expressed genes

**Figure 1:**
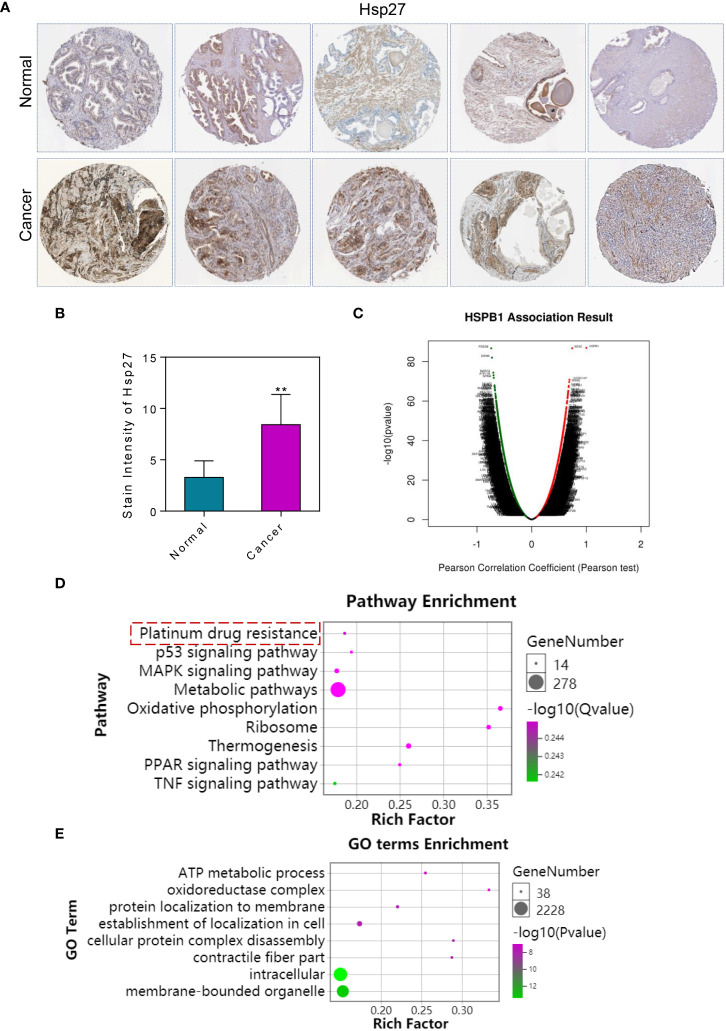
Expression of Hsp27 in prostate cancer and functional enrichment analysis of its co-expressed genes. **(A)** The expression changes of Hsp27 were analyzed using the human protein atlas database. Immunohistochemistry showed high expression of Hsp27 in the cytoplasm of tumor cells. 4×. **(B)** Immunohistochemical expression of Hsp27 in prostate cancer. **(C)** Analysis of co-expressed genes of Hsp27 in prostate cancer. Red indicates co-expression of positively correlated genes. Green indicates co-expression of negatively correlated genes. **(D)** KEGG enrichment analysis of Hsp27 co-expressed genes. **(E)** GO enrichment analysis of Hsp27 co-expressed genes. The results of enrichment analysis showed that Hsp27 and its co-expressed genes may be related to tumor drug resistance. **P <0.01 by two-tailed Student’s t-test.

The Human Protein Atlas database was used to analyze the expression changes of Hsp27 in paracancer tissues and prostate cancer tumor tissues. Immunohistochemical staining results showed that Hsp27 had a higher positive expression rate in prostate cancer tumor tissues compared to paracancer tissues (**Figures 1A, B**). Co-expressed genes of Hsp27 in prostate cancer were analyzed by LinkedOmics database website (**Figure 1C**). GO and KEGG functional enrichment analysis of Hsp27 co-expressed genes were further analyzed. The results of functional enrichment analysis showed that the pathway functions of *Hsp27* co-expressed positively related genes were mainly enriched in tumor drug resistance, oxidative phosphorylation, and cell metabolism (**Figure 1D**). GO enrichment analysis showed that *Hsp27* co-expression positively correlated genes mainly affected cell energy metabolism process, cell stress fiber bundle, cell localization, etc. (**Figure 1E**).

### 3.2 The single-cell sequencing results of *Hsp27* in prostate tissue

**Figure 2:**
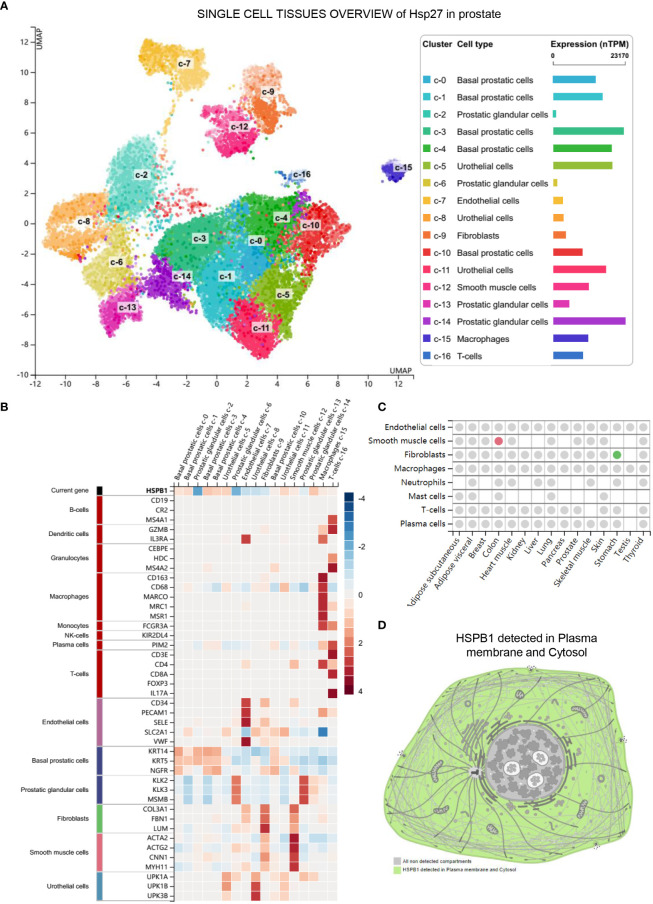
The single-cell sequencing results of Hsp27 in prostate tissue. **(A)** Expression of Hsp27 in different cells. **(B)** Cell functions involved by Hsp27. **(C)** Hsp27 affects the functions of Fibroblasts. The analysis results showed that Hsp27 may be related to tumorigenesis and development. **(D)** Hsp27 (HSPB1) detected in Plasma membrane and Cytosol.

The results showed that *Hsp27* was mainly expressed in Prostatic glandular cells, Basal prostatic cells and Urothelial cells (**Figure 2A**). The results in **Figure 2B** showed that *Hsp27* was widely involved in a variety of cellular functions. Including B-cells, macrophages, T-cells, NK-cells and so on. The results in **Figure 2C** show that *Hsp27* also affects the function of fibroblasts. **Figure 2D** shows that *Hsp27* is mainly expressed in the cytoplasm and also affects the cytoskeleton.

### 3.3 Effects of Hsp27 overexpression on DU145 and PC-3 cell survival and apoptosis

**Figure 3:**
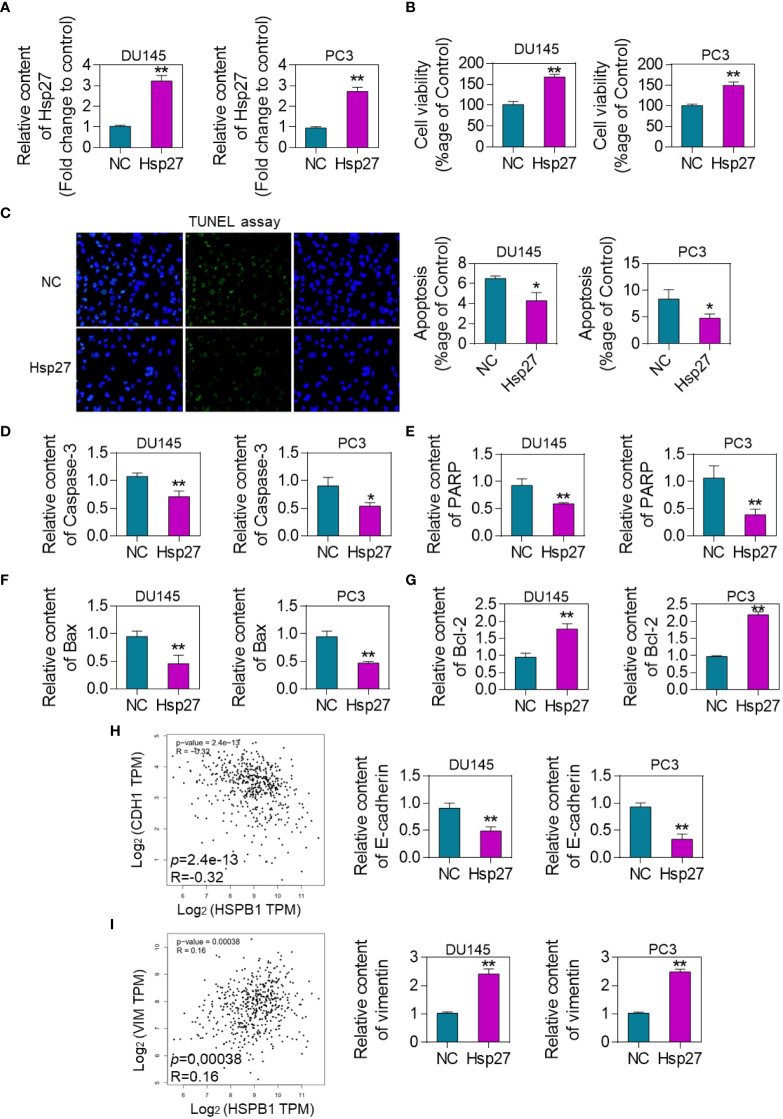
Effects of Hsp27 overexpression on DU145 and PC-3 cell survival and apoptosis. **(A)** qRT-PCR analysis of Hsp27 levels in DU145 and PC-3 cells stably transfected with empty vector (DU145 and PC-3 Mock) or human Hsp27 (DU145 and PC-3 Hsp27). **(B)** MTT assay to quantify the cell viability of DU145 and PC-3 cells overexpressing Hsp27 and Mock groups. **(C)**. TUNEL assay to detect the apoptosis of DU145 and PC-3 cells overexpressing Hsp27 and Mock group. Results are expressed as a percentage. 200× **(D)** qRT-PCR analysis of the expression levels of apoptosis-related gene caspase-3 in DU145 and PC-3 cells overexpressing Hsp27 and Mock cells. **(E)**. qRT-PCR analysis of the expression levels of apoptosis-related gene PARP in DU145 and PC-3 cells overexpressing Hsp27 and Mock cells. **(F)** qRT-PCR analysis of the expression levels of apoptosis-related gene Bax in DU145 and PC-3 cells overexpressing Hsp27 and Mock cells. **(G)** qRT-PCR analysis of the expression level of apoptosis-related gene Bcl-2 in DU145 and PC-3 cells overexpressing Hsp27 and Mock cells. *P < 0.05, **P <0.01 by two-tailed Student’s t-test. **(H)** qRT-PCR analysis of the expression level of E-cadherin in DU145 and PC-3 cells overexpressing Hsp27 and Mock cells. **(I)** qRT-PCR analysis of the expression level of Vimentin in DU145 and PC-3 cells overexpressing Hsp27 and Mock cells.

qRT-PCR analysis of Hsp27 levels in DU145 and PC-3 cells stably transfected with empty vector (DU145 and PC-3 Mock) or human Hsp27 (DU145 and PC-3 Hsp27). The results showed that the expression level of Hsp27 mRNA was significantly increased in the transfected cells (**Figure 3A**). Cells were treated with Hsp27 overexpression plasmid, and cell viability was detected by MTT assay. The results showed that overexpression of Hsp27 significantly enhanced DU145 and PC-3 cell activity (**Figure 3B**). To further study the effect of Hsp27 on DU145 and PC-3 cells of prostate cancer, apoptosis was detected by TUNEL assay. As showed in **Figure 3C**, the percentage of apoptotic cells in Hsp27-treated cells was significantly reduced compared with the control group. Expression levels of apoptosis-related genes caspase-3, PARP, Bax and Bcl-2 in DU145 and PC-3 cells overexpressing Hsp27 and Mock cells were analyzed by qRT-PCR. The results showed that after Hsp27 overexpression, caspase-3, PARP and Bax expression levels were decreased, while Bcl-2 expression levels were upregulated (**Figures 3D–G**). The results in **Figure 3H** showed that Hsp27 was negatively correlated with the co-expression of E-cadherin, an epithelial marker. Cell experiments also showed that overexpression of Hsp27 inhibited the expression of E-cadherin. Hsp27 was positively correlated with the co-expression of mesenchymal Vimentin. Cell assay results also showed that overexpression of Hsp27 up-regulated the expression of Vimentin (**Figure 3I**).

### 3.4 Inhibition of Hsp27 by ATL-1 reduced cell survival rate and induced apoptosis

**Figure 4:**
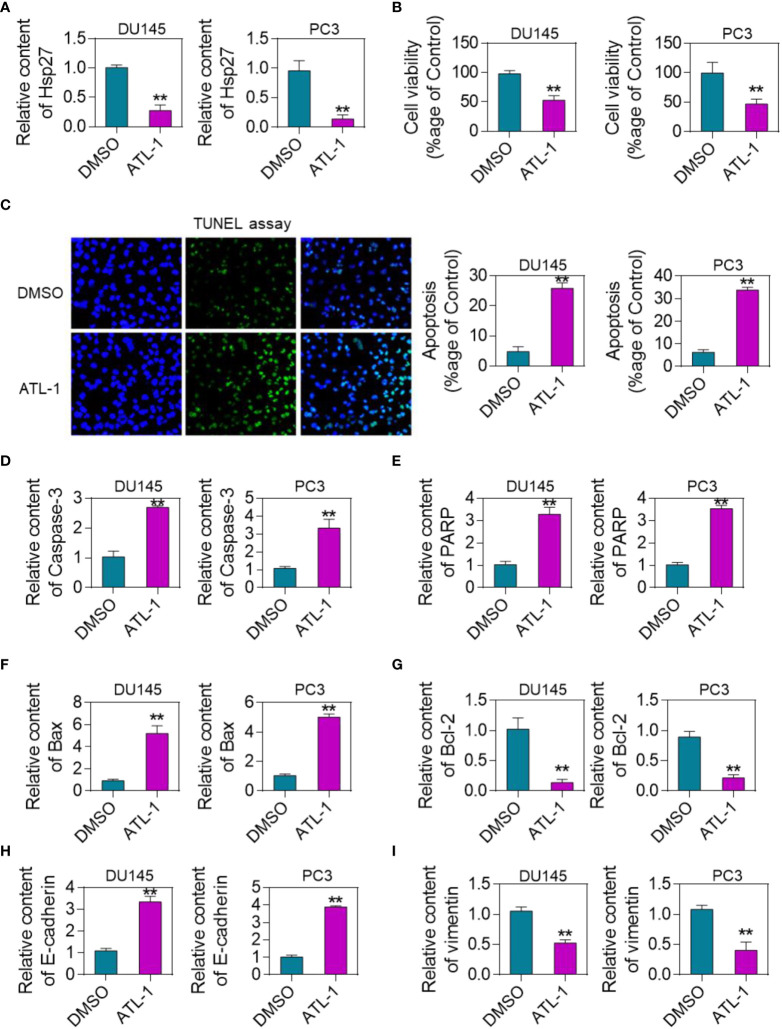
Inhibition of Hsp27 by ATL-1 reduces cell viability and induces apoptosis. **(A)** qRT-PCR analysis of Hsp27 expression levels in DU145 and PC-3 cells treated with ATL-1 or DMSO. **(B)** MTT assay to quantify the viability of ATL-1 or DMSO-treated DU145 and PC-3 cells.**(C)** TUNEL assay to detect the apoptosis of DU145 and PC-3 cells treated with ATL-1 or DMSO. Results are expressed as a percentage. 200× **(D)** qRT-PCR analysis of the expression level of apoptosis-related gene caspase-3 in DU145 and PC-3 cells treated with ATL-1 or DMSO. **(E)** qRT-PCR analysis of the expression levels of apoptosis-related gene PARP in ATL-1 or DMSO-treated DU145 and PC-3 cells. **(F)** qRT-PCR analysis of the expression level of apoptosis-related gene Bax in DU145 and PC-3 cells treated with ATL-1 or DMSO. **(G)** qRT-PCR analysis of the expression level of apoptosis-related gene Bcl-2 in DU145 and PC-3 cells treated with ATL-1 or DMSO. **P <0.01 by two-tailed Student’s t-test. **(H)** qRT-PCR analysis of the expression level of E-cadherin in DU145 and PC-3 cells treated with ATL-1 or DMSO. **(I)** qRT-PCR analysis of the expression level of Vimentin in DU145 and PC-3 cells treated with ATL-1 or DMSO.

Combined with these results, it is speculated that Hsp27 plays an important role in the malignant evolution of DU145 and PC-3 cells. The expression level of Hsp27 was detected by qRT-PCR. In cells treated with ATL-1, Hsp27 expression was down-regulated (**Figure 4A**). After treating human prostate cancer DU145 and PC-3 cells with ATL-1 for 72h, cell viability was detected by MTT assay. The results showed that ATL-1 could significantly inhibit DU145 and PC-3 cell activity (**Figure 4B**). Apoptosis of DU145 and PC-3 cells was detected by TUNEL assay. There were almost no apoptotic cells in the control group. Compared with the control group, the number of apoptotic DU145 and PC-3 cells in ATL-1 treatment group was significantly increased (**Figure 4C**). In addition, further detection showed that after ATL-1 treatment, the expression levels of apoptosis-related genes caspase-3, PARP and Bax were upregulated (**Figures 4D–F**). The expression level of anti-apoptotic gene Bcl-2 decreased after ATL-1 treatment (**Figure 4G**). These results indicated that ATL-1 could inhibit proliferation and induce apoptosis of DU145 and PC-3 cells through Hsp27. Further, we analyzed the effect of ATL-1 on EMT markers. The results of cell experiments showed that the expression of E-cadherin was up-regulated in DU145 and PC-3 cells after ATL-1 treatment (**Figure 4H**), while the expression of Vimentin was down-regulated (**Figure 4I**).

### 3.5 ATL-1 inhibition of Hsp27 enhances the antitumor effect of cabozantinib

**Figure 5:**
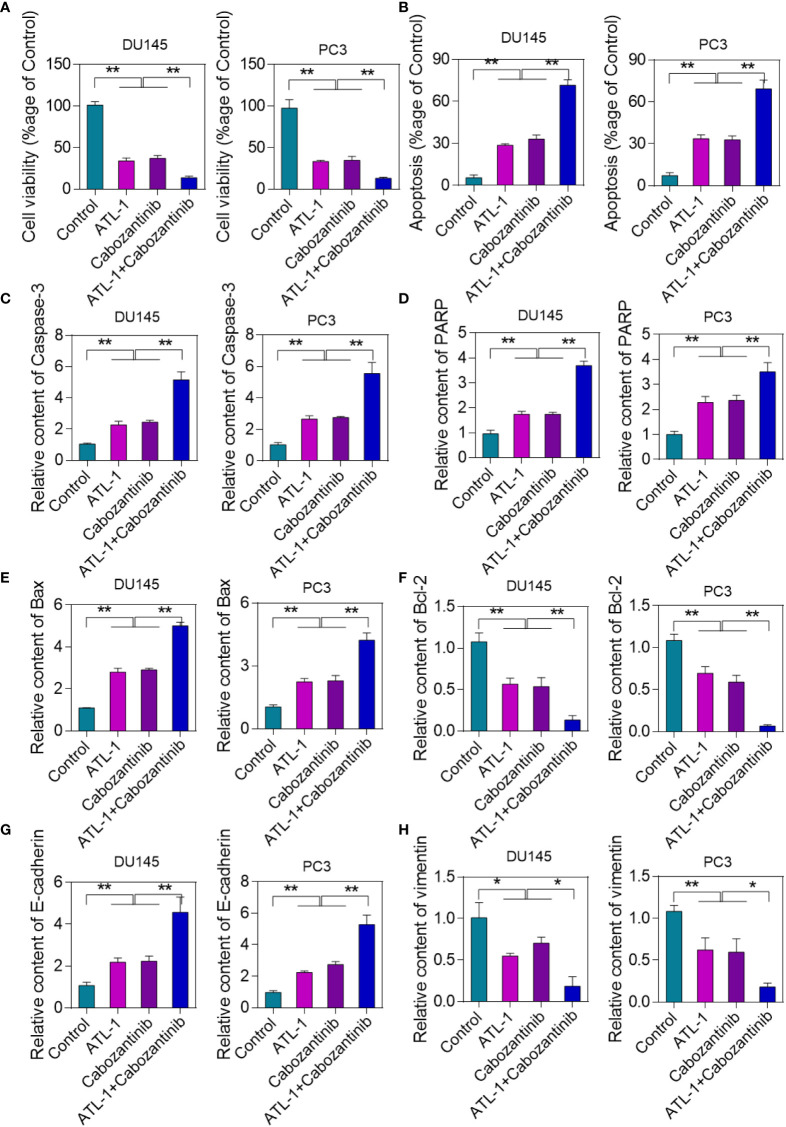
ATL-1 inhibition of Hsp27 enhances the antitumor effect of cabozantinib. **(A)** MTT assay to analyze cell viability in different treatment groups. The drug concentration of ATL-1 used was 10 μM. **(B)** TUNEL assay to detect cell apoptosis. Results are expressed as a percentage. **(C)** qRT-PCR analysis of the expression level of apoptosis-related gene caspase-3. **(D)** qRT-PCR analysis of the expression level of apoptosis-related gene PARP. **(E)** qRT-PCR analysis of the expression level of apoptosis-related gene Bax. **(F)** qRT-PCR analysis of the expression level of apoptosis-related gene Bcl-2. *p < 0.05, **p < 0.01 by one-way ANOVA analysis. **(G)** qRT-PCR analysis of the expression level of E-cadherin. **(H)** qRT-PCR analysis of the expression level of Vimenrin.

Drug combination has the advantage of reducing the side effects of chemotherapy drugs. This project analyzed whether ATL-1 had synergistic effects with cabozantinib against tumor. MTT assay showed that both ATL-1 and cabozantinib inhibited DU145 and PC-3 cell activity. However, cell activity decreased to the highest degree after the combination of the ATL-1 and cabozantinib treated group (**Figure 5A**). Cell apoptosis results showed that the combined ATL-1 and cabozantinib group had the highest cell apoptosis rate (**Figure 5B**). Apoptosis-related gene detection results showed that caspase-3, PARP and Bax expression levels were the highest in ATL-1+cabozantinib group (**Figures 5C–E**). The expression level of Bcl-2 was the lowest in ATL-1+cabozantinib group (**Figure 5F**). ATL-1 combined with cabozantinib significantly upregulated E-cadherin and inhibited Vimentin expression (**Figures 5G, H**).

### 3.6 Hsp27 regulates eIF4E and mediates cell protection

**Figure 6:**
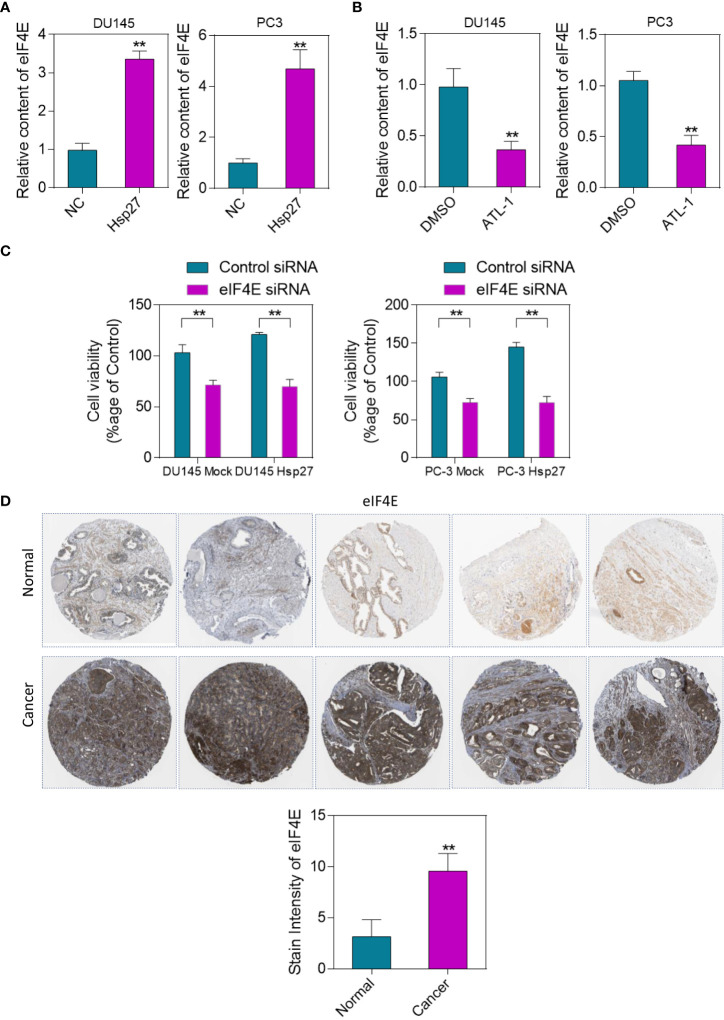
Hsp27 regulates eIF4E, mediating cytoprotective effects. **(A)** PCR analysis of eIF4E levels in DU145 and PC-3 cells stably transfected with empty vector (DU145 and PC-3 Mock) or human Hsp27 (DU145 and PC-3 Hsp27). **(B)** qRT-PCR analysis of eIF4E expression levels in DU145 and PC-3 cells treated with ATL-1 or DMSO. **(C)** Quantification of cell viability in DU145 and PC-3 Mock and DU145 and PC-3 Hsp27 cells treated with 20 nM siRNA control or eIF4E-siRNA and cabozantinib by MTT assay. **(D)** Changes of eIF4E immunohistochemical staining in human prostate cancer tissues. Immunohistochemical results showed that eIF4E was highly expressed in tumor tissues. **p < 0.01 by two-tailed Student’s t-test.

In prostate cancer cells, Hsp27 inhibits apoptosis through its regulatory effect on eIF4E. Therefore, we analyzed the effect of ATL-1 treatment on eIF4E. EIF4E levels in DU145 and PC-3 cells stably transfected with empty vector (DU145 and PC-3 Mock) or human Hsp27 (DU145 and PC-3 Hsp27) were analyzed using qRT-PCR. The results showed that eIF4E expression was up-regulated after overexpression of Hsp27 (**Figure 6A**). Subsequently, the expression level of eIF4E in DU145 and PC-3 cells treated with ATL-1 was analyzed by qRT-PCR. The results showed that the expression level of eIF4E decreased after ATL-1 treatment (**Figure 6B**). Cell viability of DU145 and PC-3 Mock and DU145 and PC-3 Hsp27 cells treated with EIF4E-SiRNA and cabozantinib was quantified by MTT assay. The results showed that Hsp27 could not change the sensitivity of cells to cabozantinib when eIF4E was silenced (**Figure 6C**). We used The Human Protein Atlas database to analyze the changes of eIF4E Immunohistochemical in human prostate cancer tissues. The results showed that eIF4E was highly expressed in human prostate cancer tumor tissues (**Figure 6D**).

### 3.7 Overexpression of EIF4E promotes drug resistance in prostate cancer

**Figure 7:**
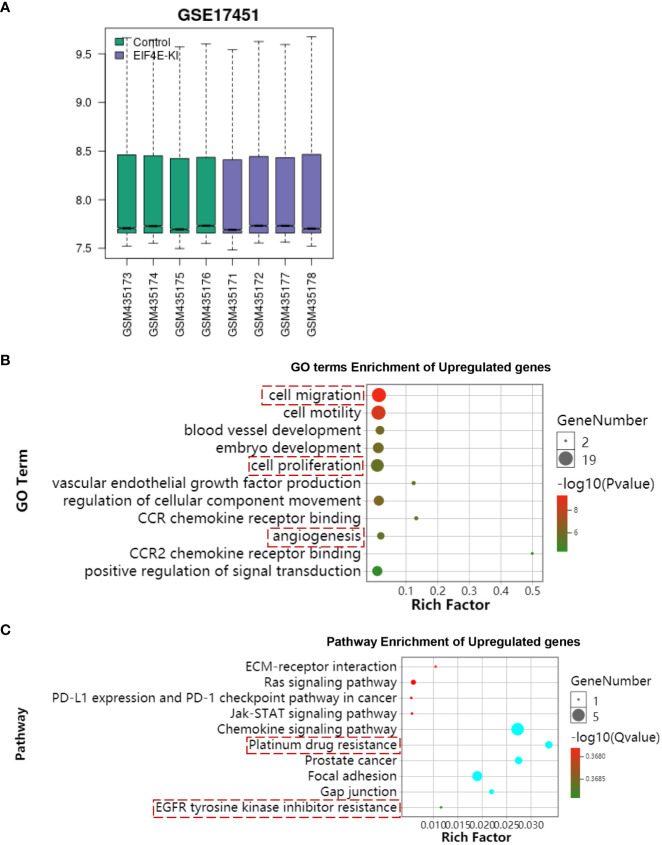
Bioinformatics analysis of overexpressed EIF4E. **(A)** The open source data of EIF4E-KI was downloaded through the GEO database. **(B)** Functional enrichment analysis of GO terms overexpressing EIF4E. **(C)** KEGG functional enrichment analysis of overexpressed EIF4E. The results of enrichment analysis showed that EIF4E overexpression was associated with tumor drug resistance.

Through the GEO database we downloaded the open source data (https://www.ncbi.nlm.nih.gov/geo/geo2r/?acc=GSE17451) of EIF4E-KI (**Figure 7A**). Further analysis of GO and KEGG functional enrichment analysis of prostate cancer after overexpression of EIF4E. The results of functional enrichment analysis showed that overexpression of EIF4E enhanced cell proliferation, cell migration, and angiogenesis (**Figure 7B**). Furthermore, prostate cancer drug resistance was enhanced after upregulation of EIF4E (**Figure 7C**). The results of this analysis are consistent with the findings of this study. This further proves the theoretical hypothesis of this study.

## 4 Discussion

Prostate cancer has a high degree of malignancy and complex pathogenesis (19). It is of great clinical significance to study the pathogenesis of prostate cancer for its diagnosis, treatment, prognosis and efficacy evaluation (20). Studies have found that many TCMs can affect the biological process of prostate cancer, and inhibit the recurrence, metastasis and prognosis of prostate cancer are closely related (21, 22).

ATL-1 belongs to sesquiterpene lactones and is one of the main active components of atractylenolide I (13). Studies have found that ATL-1 has strong anti-tumor effects on leukemia (12), bladder cancer (13) and melanoma (14). However, there are few studies on the anti-tumor effect of ATL-1 on prostate cancer, and the molecular mechanism of its anti-tumor is still unclear. The purpose of this study was to investigate the effects of ATL-1 on migration and invasion of PCa cells and its mechanism. Invasive and metastasis ability are the two basic characteristics of the malignant tumor, and PCa progress for castration resistant prostate cancer (CRPC), two important performances. ATL-1 can also inhibit cell invasion and metastasis.

TUNEL assay results showed that the positive rate of apoptotic cells in all ATL-1 treatment groups increased, indicating that ATL-1 can promote cell apoptosis, further proving the inhibitory effect of ATL-1 on PCa. Bcl-2/Bax signaling pathway is one of the classic apoptosis signals, which mediate apoptosis of many tumor cells (23, 24). In this study, the mechanism of ATL-1 regulating Bcl-2/Bax signaling pathway on tumor cells was investigated. ATL-1 may promote tumor cell apoptosis and inhibit tumor growth by regulating the expression of related proteins in Bcl-2/Bax signaling pathway. Hsp27 plays an important role in regulating the occurrence, development, metastasis, apoptosis and drug resistance of tumors, and can be used as an indicator of poor prognosis of diseases. Inhibition of Hsp27 can reverse epithelial-mesenchymal transformation (EMT) (25, 26), reduce the activity of matrix metalloproteinase (MMP), and inhibit the proliferation, migration and invasion of tumor cells (27). Hsp27 is also a target of integrin-linked kinase (ILK), which promotes the migration of bladder cancer cells (28). In addition, Hsp27 overexpression is associated with peritoneal metastasis of epithelial ovarian cancer (29). In this study, the inhibitory effect of ATL-1 on tumor metastasis is related to Hsp27 in prostate cancer cells. The expression of Hsp27 and its proliferative ability were decreased in prostate cancer cells treated with ATL-1. Subsequently, cabozantinib was tested for its inhibitory effect on prostate cancer cell lines in the presence of ATL-1. Compared with the control group, ATL-1 combined with cabozantinib reduced cell proliferation and significantly increased cell apoptosis.

Hsp27 is involved in the cell resistance of SGC7901 to 5-FU resistant strains, which is an important molecular mechanism of inducing cell multidrug resistance, and can be used as a clinical therapeutic target to improve the sensitivity of gastric cancer patients to chemotherapy (30, 31). In this study, we also found that ATL-1 targeted inhibition of heat shock protein Hsp27 enhances the antitumor effect of cabozantinib chemotherapy in prostate cancer.

## 5 Conclusion

In this study, Silence Hsp27 and ATL-1 can inhibit the proliferation of PCa and induce the apoptosis of PCa *in vitro*. ATL-1 may be promising drug in the treatment of PCa by inhibiting Hsp27. More studies are needed to elucidate how ATL-1 regulates Hsp27 expression in PCa. In addition, whether ATL-1 can also regulate Hsp27 expression *in vivo* remains to be further studied.

## Data availability statement

The datasets presented in this study can be found in online repositories. The names of the repository/repositories and accession number(s) can be found in the article/supplementary material.

## Ethics statement

Written informed consent was obtained from the individual(s) for the publication of any potentially identifiable images or data included in this article.

## Author contributions

ZT designed the study. PQ performed the experiments and analyzed the data. PQ performed the data analysis. PQ and ZT wrote the initial draft of the paper, with contributions from all authors. All authors contributed to the article and approved the submitted version.
